# Effects of Metformin on Tissue Oxidative and Dicarbonyl Stress in Transgenic Spontaneously Hypertensive Rats Expressing Human C-Reactive Protein

**DOI:** 10.1371/journal.pone.0150924

**Published:** 2016-03-10

**Authors:** Hana Malínská, Olena Oliyarnyk, Vojtěch Škop, Jan Šilhavý, Vladimír Landa, Václav Zídek, Petr Mlejnek, Miroslava Šimáková, Hynek Strnad, Ludmila Kazdová, Michal Pravenec

**Affiliations:** 1 Center for Experimental Medicine, Institute for Clinical and Experimental Medicine, Prague, Czech Republic; 2 Institute of Physiology, Czech Academy of Sciences, Prague, Czech Republic; 3 Institute of Molecular Genetics, Czech Academy of Sciences, Prague, Czech Republic; Max-Delbrück Center for Molecular Medicine (MDC), GERMANY

## Abstract

Inflammation and oxidative and dicarbonyl stress play important roles in the pathogenesis of type 2 diabetes. Metformin is the first-line drug of choice for the treatment of type 2 diabetes because it effectively suppresses gluconeogenesis in the liver. However, its “pleiotropic” effects remain controversial. In the current study, we tested the effects of metformin on inflammation, oxidative and dicarbonyl stress in an animal model of inflammation and metabolic syndrome, using spontaneously hypertensive rats that transgenically express human C-reactive protein (SHR-CRP). We treated 8-month-old male transgenic SHR-CRP rats with metformin (5 mg/kg/day) mixed as part of a standard diet for 4 weeks. A corresponding untreated control group of male transgenic SHR-CRP rats were fed a standard diet without metformin. In a similar fashion, we studied a group of nontransgenic SHR treated with metformin and an untreated group of nontransgenic SHR controls. In each group, we studied 6 animals. Parameters of glucose and lipid metabolism and oxidative and dicarbonyl stress were measured using standard methods. Gene expression profiles were determined using Affymetrix GeneChip Arrays. Statistical significance was evaluated by two-way ANOVA. In the SHR-CRP transgenic strain, we found that metformin treatment decreased circulating levels of inflammatory response marker IL-6, TNFα and MCP-1 while levels of human CRP remained unchanged. Metformin significantly reduced oxidative stress (levels of conjugated dienes and TBARS) and dicarbonyl stress (levels of methylglyoxal) in left ventricles, but not in kidneys. No significant effects of metformin on oxidative and dicarbonyl stress were observed in SHR controls. In addition, metformin treatment reduced adipose tissue lipolysis associated with human CRP. Possible molecular mechanisms of metformin action–studied by gene expression profiling in the liver–revealed deregulated genes from inflammatory and insulin signaling, AMP-activated protein kinase (AMPK) signaling and gluconeogenesis pathways. It can be concluded that in the presence of high levels of human CRP, metformin protects against inflammation and oxidative and dicarbonyl stress in the heart, but not in the kidney. Accordingly, these cardioprotective effects of metformin might be especially effective in diabetic patients with high levels of CRP.

## Introduction

It has been demonstrated that obesity and insulin resistance are associated with a proinflammatory state, which may be mediated by cytokines and subsequently cause elevated levels of CRP that might predispose to an increased risk of type 2 diabetes [1,2]. Metformin is the most widely used drug for the treatment of type 2 diabetes because it effectively suppresses gluconeogenesis in the liver. In addition, metformin treatment is associated with cardioprotective effects and reduced cardiovascular morbidity and mortality. Cardioprotective effects of metformin are, at least in part, independent of improvement in glycemic control and other risk factors. These findings raise the possibility that metformin could decrease the risk of cardiovascular disease through its pleiotropic effects [3–5]. Recent data provide evidence that inflammation, oxidative stress, the presence of lipoperoxidation products and hyperglycemia are associated with abnormal cellular accumulation of the reactive dicarbonyl metabolites that induce increased protein and DNA modification, which contribute to cell and tissue dysfunction in ageing and disease [6–8]. In addition, synthesis of dicarbonyl metabolites may be independent of persistent hyperglycemia because it is possible that these metabolites may also be produced even at normal glucose concentrations by changes in glucose levels [9]. Dicarbonyls are also produced during the course of lipid and amino acid metabolism. Furthermore, dicarbonyl levels in the organism are increased by consumption of some foodstuffs processed by grilling, dry baking or prolonged storage [10]. Increased levels of dicarbonyls are associated with increased levels of triglycerides and free fatty acids, lipid peroxidation and with the degradation of glycated proteins observed in type 1 and 2 diabetes. Methylglyoxal is increased 25 and 15 times in patients with type 1 and type 2 diabetes, respectively. The most important glycating agents are reactive dicarbonyl species, such as glyoxal (GL), methylglyoxal (MG) and 3-deoxyglucosone (3-DG). Individual dicarbonyls differ in their biosynthesis, metabolism and major protein and DNA adducts, as summarized by Rabbani et al. [11]. Dicarbonyls are characterized by their extremely high chemical reactivity. They are very reactive and thus can produce the covalent modification of proteins, lipids and nucleic acids and form advanced glycation end products (AGEs) even at very low levels. Human and animal studies provided evidence that MG plays an important role in pathologies related to AGE accumulation, including diabetic macro- and microvascular complications, such as nephropathy and cardiac failure [12]. MG is increased in obese and diabetic individuals, but less is known about levels of GL and 3-DG. Practically nothing is known about the role of tissue levels of individual dicarbonyls and their relationship to oxidative stress in the pathogenesis of diabetic complications.

Recently, we derived a new model of inflammation, metabolic disturbances and target organ damage, spontaneously hypertensive rats that express human C-reactive protein (CRP) in the liver (SHR-CRP transgenic rats) [13]. In the current study, we tested the hypothesis that metformin can protect against CRP-induced inflammation and thus ameliorate oxidative stress and metabolic disturbances. We found that metformin ameliorates insulin resistance and dyslipidemia and exhibit cardioprotective effects by reducing inflammation and tissue oxidative and dicarbonyl stress associated with human CRP.

## Results

### Effects of metformin on serum levels of human CRP and rat CRP and on markers of inflammation induced by human CRP

**Fig 1:**
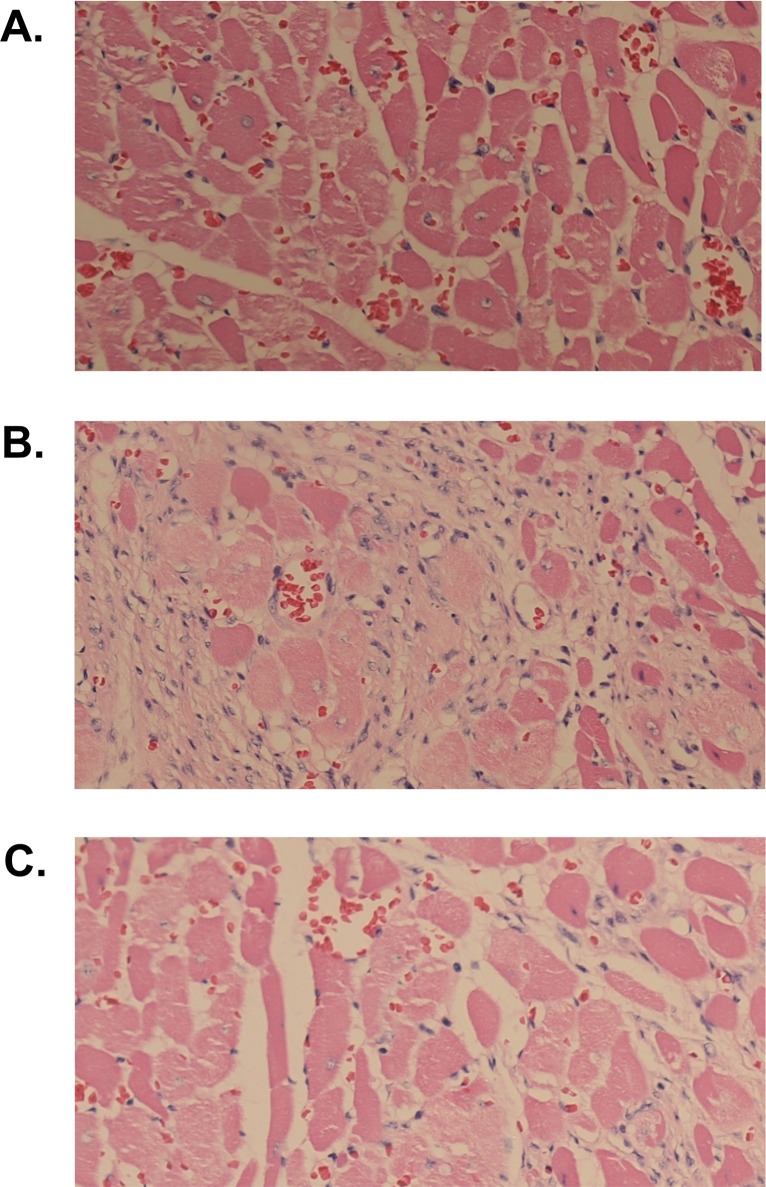
Histology. Histological examination of the heart of SHR rats treated with placebo (A), SHR-CRP rats treated with placebo (B), and SHR-CRP rats treated with metformin (C). As can be seen, SHR-CRP rats treated with metformin exhibited a marked reduction of inflammatory cellular infiltrate when compared to SHR-CRP rats treated with placebo. Magnification 20X.

As shown in Table 1, metformin had no significant effects on serum concentrations of transgenic human CRP. On the other hand, serum levels of rat endogenous CRP were significantly reduced in SHR-CRP transgenic rats and were not affected after treatment with metformin. In addition, metformin treatment was associated with a significant reduction in serum levels of IL-6, TNFα and MCP-1 while no significant decreases of these markers were observed in nontransgenic SHR controls. Fig 1 shows inflammatory infiltration in the heart. As can be seen, SHR-CRP rats treated with placebo exhibited a marked inflammatory infiltration (Fig 1B) when compared to SHR-CRP rats treated with metformin (Fig 1C) and to SHR controls (Fig 1A).

**Table 1:** Biochemical and metabolic parameters in SHR-CRP transgenic and wild type controls treated with metformin or placebo.

Trait	SHR placebo	SHR metformin	SHR-CRP placebo	SHR-CRP metformin
Body weight (g)^a^	403±11^f^	362±4^d^	360±15	361±7
Relative weight of epididymal fat (g/100 g body weight)^a^	1.27±0.04^f^	0.92±0.06^d^	0.85±0.07	0.79±0.04
Relative weight of interscapular BAT (g/100 g body weight)^a^	0.061±0.004	0.069±0.03	0.051±0.004	0.045±0.003
Relative liver weight (g/100 g body weight)^a^	3.31±0.06	3.37±0.05	3.51±0.06	3.25±0.10^e^
Serum glucose (mmol/L)	6.4±0.3	6.9±0.1	6.6±0.2	6.2±0.2
Serum insulin (nmol/L)^c^	0.304±0.081	0.157±0.033	0.403±0.067	0.189±0.042
Serum triglycerides (mmol/L)^b^^c^	0.89±0.04	0.66±0.05	1.12±0.11	0.85±0.02
Serum NEFA (mmol/L)	0.70±0.06	0.53±0.03	0.69±0.09	0.64±0.07
Serum cholesterol (mmol/L)^c^	1.66±0.06	1.50±0.07	1.65±0.10	1.49±0.02
Serum IL-6 (pg/ml)^a^	9.8±1.3^f^	7.6±1.0	15.6±1.5	7.8±1.4^e^
Serum TNFα (pg/ml)^a^	2.04±0.08^f^	2.07±0.06	2.69±0.16	1.47±0.44^e^
Serum MCP-1 (ng/ml)^a^	9.3±0.8^f^	9.0±1.9	13.9±0.9	9.1±0.9^e^
Rat CRP (μg/ml)^b^	233±11	208±12	75±5	66±4
Human CRP (μg/ml)^b^	n.d.	n.d.	633±18	649±26
Liver triglycerides (μmol/g)^c^	9.3±0.5	7.4±0.3	9.3±0.9	6.7±0.5
Basal lipogenesis in WAT (nmol gl./mg prot./2 h)^b^^c^	111±9	185±11	82±12	136±13
Insulin-stimulated lipogenesis in WAT (nmol gl./mg prot./2 h)^c^	208±19	296±23	213±20	263±30
Basal glycogenesis (nmol gl./g/2 h)^b^^c^	86±6	129±20	136±13	180±10
Insulin stimulated glycogenesis (nmol gl./g/2 h)^b^^c^	228±20	355±34	185±15	258±28
Glucose oxidation in muscle (nmol gl./mg/2 h)^b^^c^	354±12	382±32	236±10	303±15
Basal lipolysis (NEFA μmol/g)^a^	3.57±0.50^f^	3.35±0.42	8.52±0.92	4.04±0.70^e^
Adrenaline-stimulated lipolysis (NEFA μmol/g)^a^	6.03±0.66^f^	7.95±0.66	12.07±1.16	6.89±0.99^e^

Two-way ANOVA results:

^a^ denotes significant P <0.05 metformin treatment x human CRP interaction (treatment x strain comparison)–metformin treatment can protect against adverse effects that are dependent on human CRP.

^b^ denotes P<0.05 significance of SHR-CRP vs. SHR controls (strain effects)

^c^ denotes P<0.05 significance of metformin treatment vs. placebo (treatment effects). For comparisons versus controls, the Holm-Sidak test was used

^d^ denotes P<0.05 significance of comparisons for metformin vs. placebo treatment within nontransgenic SHR

^e^ denotes P<0.05 significance of comparisons for metformin vs. placebo treatment within transgenic SHR-CRP

^f^ denotes P<0.05 significance of comparisons of SHR vs. SHR-CRP treated with placebo; n.d. denotes not detected.

### Effects of metformin on oxidative stress-related parameters

As shown in Table 2, metformin treatment of SHR-CRP transgenic rats was associated with a significant reduction of oxidative stress (lower levels of conjugated dienes and TBARS) in the liver and left ventricles, while metformin treatment did not affect oxidative stress in the liver and left ventricles in nontransgenic controls. On the other hand, metformin treatment had no significant effect on parameters of oxidative stress in the kidney cortex. In addition, the activities of antioxidative enzymes associated with human CRP were not affected by metformin treatment (Table 2).

**Table 2:** Parameters of oxidative stress in the liver in SHR-CRP transgenic and wild type controls treated with metformin or placebo.

Trait	SHR placebo	SHR metformin	SHR-CRP placebo	SHR-CRP metformin
**Liver**				
SOD (U/mg)^b^	0.155±0.13	0.151±0.11	0.118±0.005	0.120±0.006
GSH-Px (μM NADPH/min/mg)^c^	302±16	318±21	240±17	318±19
GR (μM NADPH/min/mg)	135±9	132±7	139±12	133±5
CAT (mM H_2_O_2_/min/mg)^b^	1351±96	1377±84	1723±63	1924±40
GSH (μM/mg prot.)	61.8±9.4	48.8±1.6	55.1±2.9	58.8±1.5
Conjugated dienes (nM/mg)^a^	42.9±1.3^f^	41±2.3	53.9±1.9	43±2^e^
TBARS (nM/mg)^a^	1.279±0.082^f^	1.165±0.042	1.668±0.107	1.185±0.084^e^
**Left ventricle**				
SOD (U/mg)	0.051±0.003	0.045±0.004	0.045±0.003	0.049±0.004
GSH-Px (μM NADPH/min/mg)^b^^c^	177±11	228±19	149±15	166±8
GR (μM NADPH/min/mg)^b^^c^	74±5	56±5	50±4	58±5
CAT (mM H_2_O_2_/min/mg)	609±44	520±30	513±45	553±49
GSH (μM/mg prot.)^b^^c^	22.6±0.7	26.6±1.5	20.7±0.8	23.6±0.8
Conjugated dienes (nM/mg)^a^	22.9±0.8^f^	24.0±1.2	28.1±1.2	23.3±0.3^e^
TBARS (nM/mg)^a^	0.771±0.039^f^	0.746±0.041	0.921±0.043	0.653±0.044^e^
**Kidney cortex**				
SOD (U/mg)^b^^c^	0.084±0.004	0.096±0.005	0.048±0.005	0.063±0.005
GSH-Px (μM NADPH/min/mg)	160±16	173±21	136±10	161±13
GR (μM NADPH/min/mg)^b^^c^	44±2	61±2	31±3	40±5
CAT (mM H_2_O_2_/min/mg)	697±63	664±61	711±48	722±61
GSH (μM/mg prot.)	20.4±1.6	17.9±1.7	17.8±1.3	19.5±1
Conjugated dienes (nM/mg)	28.3±3.3	24.5±1.5	31.6±2.8	27.1±1.2
TBARS (nM/mg)	0.669±0.074	0.579±0.045	0.654±0.032	0.564±0.027

Two-way ANOVA results

^a^ denotes significant P <0.05 metformin treatment x human CRP interaction (treatment x strain comparison)–metformin treatment can protect against oxidative stress that is dependent on human CRP.

^b^ denotes P<0.05 significance of SHR-CRP vs. SHR controls (strain effects)

^c^ denotes P<0.05 significance of metformin treatment vs. placebo (treatment effects). For comparisons versus controls, the Holm-Sidak test was used

^e^ denotes P<0.05 significance of comparisons for metformin vs. placebo treatment within transgenic SHR-CRP

^f^ denotes P<0.05 significance of comparisons of SHR vs. SHR-CRP treated with placebo.

### Effects of metformin on dicarbonyl stress-related parameters

**Fig 2:**
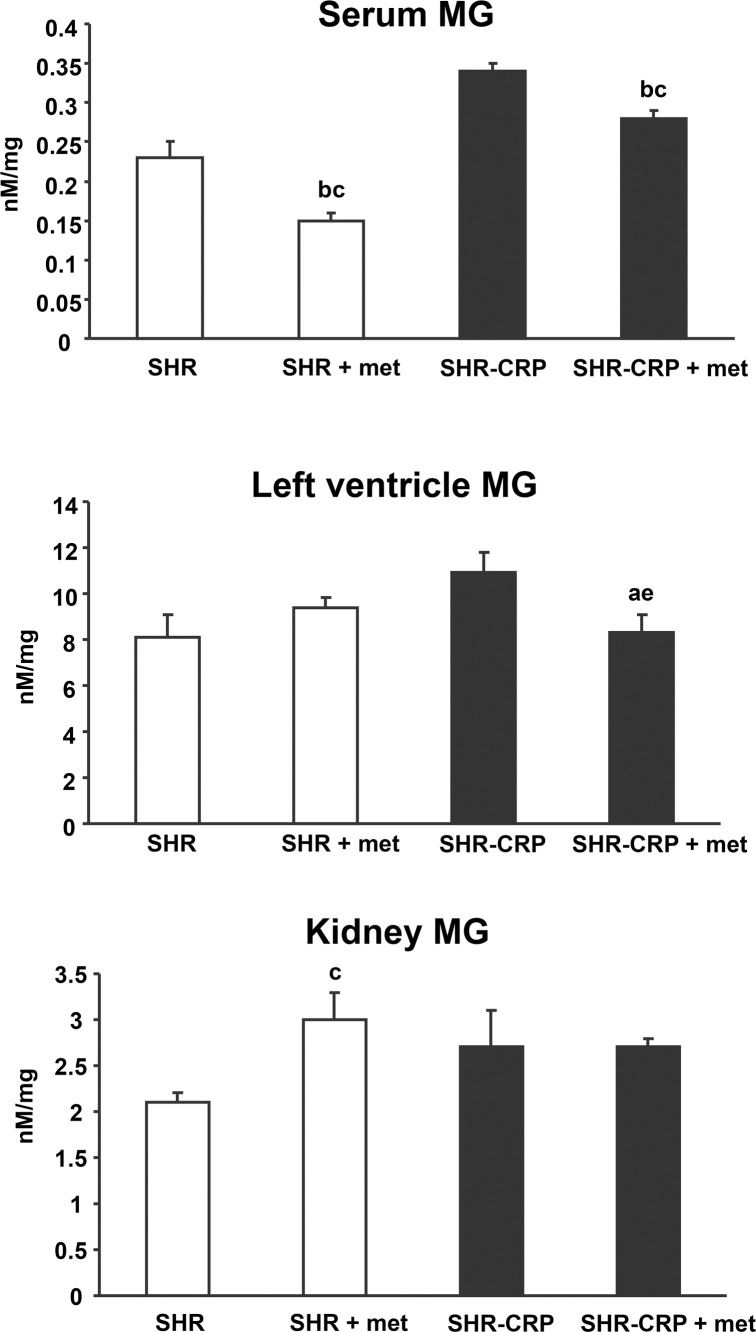
Serum and tissue methylglyoxal (MG). MG levels in left ventricles isolated from transgenic SHR-CRP rats treated with metformin were significantly reduced when compared to untreated SHR-CRP controls, while no effects of metformin were observed in nontransgenic SHR. No effects of metformin on MG levels were observed in kidneys isolated from SHR transgenic rats when compared to untreated SHR-CRP. Two-way ANOVA results: ^a^ denotes significant P <0.05 metformin treatment x human CRP interaction (treatment x strain comparison)–metformin treatment can protect against adverse effects that are dependent on human CRP. ^b^ denotes P<0.05 significance of SHR-CRP vs. SHR controls (strain effects); ^c^ denotes P<0.05 significance of metformin treatment vs. placebo (treatment effects). For comparisons versus controls, the Holm-Sidak test was used: ^e^ denotes P<0.05 significance of comparisons for metformin vs. placebo treatment within transgenic SHR-CRP.

As shown in Fig 2 and Table 3, metformin protected cardiac left ventricles against dicarbonyl stress associated with human CRP when MG levels were significantly reduced in SHR-CRP rats treated with metformin versus untreated SHR-CRP. On the other hand, no protective effects of metformin were observed in kidneys. Serum DG, MG and 3-DG concentrations did not correlate with their respective tissue levels (data not shown).

**Table 3:** Parameters of dicarbonyl stress.

Trait	SHR placebo	SHR metformin	SHR-CRP placebo	SHR-CRP metformin
Serum methylglyoxal (nM/mg)^b^^c^	0.234±0.015	0.151±0.009	0.342±0.007	0.281±0.014
Serum glyoxal (nM/mg)^b^	0.193±0.009	0.178±0.01	0.238±0.015	0.227±0.008
Serum 3-deoxyglucosone (nM/mg)^c^	0.0405±0.0004	0.0344±0.0014	0.0476±0.0031	0.0358±0.0043
Left ventricle methylglyoxal (nM/mg)^a^	8.086±1^f^	9.443±0.402	10.912±0.897	8.364±0.853^e^
Left ventricular glyoxal (nM/mg)^b^^c^	3.173±0.077	3.614±0.255	3.784±0.209	4.302±0.024
Left ventricular 3-deoxyglucosone (nM/mg)	0.867±0.053	0.899±0.127	0.99±0.031	0.857±0.026
Kidney methylglyoxal (nM/mg)^c^	2.132±0.081	3.043±0.286	2.669±0.36	2.684±0.089
Kidney glyoxal (nM/mg)^b^	0.604±0.066	0.605±0.081	0.955±0.059	0.997±0.04
Kidney 3-deoxyglucosone (nM/mg)	0.576±0.127	0.605±0.054	0.683±0.067	0.596±0.107

Two-way ANOVA results

^a^ denotes significant P <0.05 metformin treatment x human CRP interaction (treatment x strain comparison)–metformin treatment can protect against oxidative stress that is dependent on human CRP.

^b^ denotes P<0.05 significance of SHR-CRP vs. SHR controls (strain effects)

^c^ denotes P<0.05 significance of metformin treatment vs. placebo (treatment effects). For comparisons versus controls, the Holm-Sidak test was used

^e^ denotes P<0.05 significance of comparisons for metformin vs. placebo treatment within transgenic SHR-CRP

^f^ denotes P<0.05 significance of comparisons of SHR vs. SHR-CRP treated with placebo; n.d. denotes not detected.

### Metabolic effects of metformin

Metformin treatment was associated with a small but significant reduction in body weight and relative epididymal fat weight and interscapular brown adipose tissue (BAT) weight in the nontransgenic strain, while there were no significant differences in the transgenic SHR-CRP strain (Table 1). Transgenic rats, but not nontransgenic controls, treated with metformin exhibited significantly reduced relative liver weights when compared to their respective untreated controls (Table 1). Significant treatment effects were observed when serum insulin, triglycerides and cholesterol levels and liver triglyceride levels were reduced and sensitivity of muscle and adipose tissue to insulin action was increased after metformin treatment in both SHR-CRP transgenic rats and nontransgenic controls. On the other hand, there were significant strain x treatment interactions when both basal- and adrenaline-stimulated lipolysis in white adipose tissue were reduced in metformin-treated SHR-CRP transgenic rats, while no significant effects were observed in nontransgenic controls (Table 1).

### Gene expression profiling

To search for responsible molecular mechanisms of the effects of metformin on inflammation, oxidative and dicarbonyl stress and metabolic disturbances associated with transgenic expression of human CRP, we performed genome-wide expression profiling in the liver isolated from SHR and SHR-CRP strains treated with metformin or a placebo. Altogether, we detected 51 genes showing significant differential expression (q<0.05) (S2 Table) and 2864 genes were differentially expressed at a nominal p<0.05 value. The directional expression of selected genes was confirmed using real time PCR (S1 Fig). Effects of metformin on hepatic gene expression levels in the SHR-CRP transgenic strain were similar to those in the nontransgenic strain. Although we did not detect any significant treatment x strain interactions, we observed significant metformin treatment effects. Table 4 displays genes from KEGG pathways identified by SPIA, including circadian rhythms, insulin signaling, maturity onset diabetes of the young, and type 2 diabetes pathways. These pathways include deregulated genes from AMP-activated protein kinase (AMPK) (*Prkaa1*, *Prkab2*, *Ppargc1a*) and gluconeogenesis pathways (*Pck1*, *G6pc3*, *Ppp1rc3*). The influenza A pathway includes cytosolic DNA-sensing pathway genes (*Ddx58*, *Casp1*, *Adar*, *Cxcl10*, *Irf7*, *IL-6*, *Nfkb1*), toll-like receptor signaling pathway genes (*Jun*, *Cxcl10*, *Ifnar1*, *Irf7*, *IL-6*, *Irak4*, *Nfkb1*, *Pik3r3*), RIG-I-like receptor signaling pathway genes (*Ddx58*, *Cxcl10*, *Ifih1*, *Irf7*, *Nfkb1*, *Trim25*), cytokine-cytokine receptor interaction pathway genes (*Fas*, *Ccl12*, *Ccl2*, *Cxcl10*, *Ifnar1*, *Ifngr2*, *IL-6*, *Tnfsf10*), natural killer cell-mediated cytotoxicity pathway genes (*Fas*, *Icam1*, *Ifnar1*, *Ifngr2*, *Pik3r3*, *Tnfsf10*), NOD-like receptor signaling pathway genes (*Casp1*, *Ccl12*, *Ccl2*, *IL-6*, *Nfkb1*), chemokine signaling pathway genes (*Ccl12*, *Ccl2*, *Cxcl10*, *Nfkb1*, *Pik3r3*) and apoptosis pathway genes (*Fas*, *Irak4*, *Nfkb1*, *Pik3r3*, *Tnfsf10*). Practically all these genes are downregulated after treatment with metformin, which supports the anti-inflammatory effects of metformin. In addition, we observed differential expression of the JAK-STAT pathway, which is the main intracellular cascade initiated in response to cytokine signaling. Transcriptional misregulation in the cancer pathway included deregulated genes, which play an important role in cell cycle (*Zbtb16*, *Ccnt2*) and inflammatory processes (*Nfkbiz*, *IL-6*, *Nfkb1*, *Birc3*, *Tmprss2*).

**Table 4:** List of genes from KEGG pathways identified by SPIA showing the effects of metformin versus placebo in SHR controls (A) and in SHR-CRP rats (B).

**A. Effects of metformin vs. placebo in SHR controls**	**FWER**	**Deregulated genes (P<0.05)**
Circadian rhythms	2.57e-06	↓*Npas2*, ↑*Per1*, ↑*Per2*, ↑*Per3*, ↑*Nr1d1*, ↓*Arnlt*, ↑*Cry2*, ↑*Prkab2*
Insulin signaling	0.0028	↓*Socs2*, ↑*Foxo1*, ↑*Ppargc1a*, ↑*Cblb*, ↓*Ptpn1*, ↑*Mapk3*, ↑*Mknk2*, ↑*Calm2*, ↓*G6pc3*, ↓*Irs3*, ↓*Akt1*, ↑*Prkab2*
Maturity onset diabetes of the young	0.012	↑*Hes1*, ↓*Onecut1*, ↓*Hnf4a*, ↓*Hhex*, ↑*Nr5a2*, ↓*Iapp*, ↑*Slc2a2*
**B. Effects of metformin vs. placebo in SHR-CRP rats**	**FWER**	**Deregulated genes (P<0.05)**
Circadian rhythms	1.01e-05	↑*Per2*, ↑*Per1*, ↓*Npas2*, ↓*Prkaa1*, ↑*Nr1d1*, ↑*Per3*, ↓*Cul1*, ↑*Bhlhe40*, ↓*Arnlt*, ↑*Csnk1d*
JAK-STAT signaling	0.00004	↑*Il6r*, ↓*Socs2*, ↓*Cish*, ↑*Spry4*, ↓*Osmr*, ↓*Il13ra1*, ↓*Il6*, ↑*Cblb*, ↓*Il7*, ↓*Stam2*, ↓*Ifngr2*, ↓*Myc*, ↓*Ifnar1*, ↓*Irf9*, ↑*Epor*, ↑*Spred1*, ↓*Csf2rb*, ↑*Bcl2l1*, ↑*Socs1*
Influenza A	0.02	↓*Fas*, ↓*Icam1*, ↓*Mx2*, ↓*Irf7*, ↓*Kpna2*, ↓*Cxcl10*, ↓*Il6*, ↓*Ifih1*, ↓*Ccl12*, ↓*Rsad2*, ↓*Rae1*, ↓*Ccl2*, ↓*Ddx58*, ↓*Tnfsf10*, ↓*Nfkb1*, ↓*Jun*, ↑*Tmprss13*, ↓*Ifngr2*, ↓*Ifnar1*, ↓*Casp1*, ↓*Oas1b*, ↓*Trim25*, ↓*Irak4*, ↓*Irf9*, ↓*Tmprss2*, ↓*Adar*, ↓*Pik3r3*, ↓*Nxt1*
Maturity onset diabetes of the young	0.014	↓*Hhex*, ↓*Onecut1*, ↑*Hes1*, ↓*Ins2*, ↑*Nr5a2*
Insulin signaling	0.011	↓*Socs2*, ↑*Foxo1*, ↓*Prkaa1*, ↑*Ppargc1a*, ↑*Cblb*, ↑*Mknk2*, ↓*Nras*, ↑*Ppp1r3c*, ↓*Ins2*, ↑*Irs2*, ↑*Socs1*, ↑*Pck1*
Transcriptional misregulation in cancer	0.011	↑*Zbtb16*, ↑*Bcl6*, ↑*Per2*, ↑*Foxo1*, ↓*Hhex*, ↓*Hist1h2bb*, ↑*Ccnt2*, ↑*Nfkbiz*, ↓*Ss18*, ↓*Il6*, ↓*H3f3b*, ↑*Rxra*, ↓*Spi1*, ↓*Meis1*, ↓*Nfkb1*, ↓*Myc*, ↓*Jmjd1c*, ↑*Slc45a3*, ↓*Tmprss2*, ↑*Etv4*, ↓*Pdgfa*, ↑*Bcl2l1*, ↓*Birc3*, ↑*Pbx1*, ↓*Fcgr1a*, ↑*Runx2*
Type II diabetes	0.043	↓*Socs2*, ↓*Ins2*, ↑*Irs2*, ↑*Socs1*

↑ and ↓ denote up- and downregulated, respectively, in metformin- versus placebo-treated rats. FWER–Family-Wise Error Rate

## Discussion

In the current study, we found that metformin can attenuate inflammation and tissue oxidative and dicarbonyl stress which are all dependent on human CRP. In addition, we report the novel finding that in the presence of increased levels of human CRP, metformin can reduce dicarbonyl stress in the heart, whereas no effects of metformin were observed in the kidney. Metformin treatment was associated with significant reduction of rat CRP. As can be seen in Table 4, SHR-CRP rats treated with metformin had reduced expression of *Il6* gene which might explain reduced levels of rat serum IL-6 and CRP.

In human clinical trials in patients with type 2 diabetes, metformin treatment was associated with both significantly reduced plasma MG and markers of oxidative stress [14,15]. On the other hand, there are no reports on dicarbonyl stress at organ level in humans. Results of the current study show that metformin treatment can ameliorate dicarbonyl stress in the heart, but not in the kidney. It is interesting that the protective effects of metformin (in the presence of transgenic human CRP) on oxidative stress were significant in the heart but absent in the kidney, and were congruent with protective effects of metformin on dicarbonyl stress. It is, therefore, possible that reduced dicarbonyl levels in the heart are secondary to reduced oxidative stress. Chronic inflammation associated with the expression of the human CRP transgene may cause neutrofils, eosinofils and macrophages to increase production of reactive oxygen species (ROS) [16]. Increased levels of ROS induce oxidative stress and, consequently, dicarbonyl stress. Metformin reduces inflammation and oxidative and dicarbonyl stress in turn. In addition, metformin is a mild inhibitor of mitochondrial respiratory chain complex I and thus also contributes to reduced ROS production [17]. In the current study, we observed no effects of metformin treatment on expression of the *Glo1* gene, which suggests that metformin reduces dicarbonyl stress more as a result of its anti-inflammatory effects than by increasing activity of *Glo1* [14]. It should be noted that we observed no significant correlations between serum MG and tissue MG levels. Accordingly, serum MG levels in humans must be regarded with caution as a marker of tissue dicarbonyl stress.

As can be seen in Table 1, metformin had no significant effects on glucose levels. Both the SHR-CRP and SHR strains are normoglycemic and it is likely that these rats were protected against metformin-induced hypoglycemia. On the other hand, metformin had significant treatment effects on insulin when insulin levels were significantly reduced in both transgenic and control rats.

To search for responsible molecular mechanisms of the effects of metformin we performed a genome-wide expression analysis and found significant effects of metformin treatment on hepatic gene expression levels in both the SHR-CRP transgenic strain and the nontransgenic strain. For example, metformin treatment was associated with reduced expression of genes related to innate immune response pathways and inflammation, including cytosolic DNA-sensing, toll-like receptor signaling, RIG-I-like receptor signaling, cytokine-cytokine receptor interaction, natural killer cell-mediated cytotoxicity, NOD-like receptor signaling and chemokine signaling. In mammals, four Janus kinases (JAK) and seven STAT family members mediate the action of almost 40 cytokines receptors. As shown in Table 4, metformin treatment was associated mostly with reduced expression of genes from the JAK-STAT signaling pathway. These results provide compelling evidence that metformin protects against adverse effects of human CRP by reducing expression of genes involved in innate immune response and inflammation.

Metabolic pathways included deregulated genes from the AMP-activated protein kinase (AMPK) pathway, gluconeogenesis as well as genes from insulin signaling, type II diabetes signaling, maturity onset diabetes of the young and circadian rhythm pathways. Interactions between metformin and polymorphisms of some of the abovementioned genes (human PRKAA1, PRKAB2, PPRGC1A, PCK1, HNF4A, CRY2) have been reported in human studies [18]. Metformin activates AMP-activated protein kinase (AMPK) in hepatocytes. AMPK affects diverse metabolic pathways including gluconeogenesis and fatty acid metabolism. In addition, AMPK blocks NFKB activation and reduces IL-6 release, thereby decreasing inflammation (both *Nfkb1* and *IL6* hepatic expression are reduced) [5].

Metformin treatment is also associated with deregulation of genes from the circadian rhythm pathway. It has been suggested that metformin partially inhibits mitochondrial complex I, which leads to increased NADH and reduced ATP levels. The decrease in the NAD^+^/NADH ratio after metformin treatment results in CLOCK-BMAL1 promoter binding and increased CLOCK-BMAL1-mediated transcription. In turn, elevated levels of PPARγ and PGC1α (*Ppargc1a* increased expression in the current study) increase β-oxidation [19].

In summary, our results provide compelling evidence for the pleiotropic effects of metformin, including protection against inflammation and oxidative and dicarbonyl stress in the heart associated with the expression of human CRP. It can be concluded that (1) metformin cardioprotection might be especially effective in diabetic patients with high levels of CRP and (2) that the lack of significant correlations between serum and tissue MG levels strongly suggests that serum MG levels might not be reliable parameters of dicarbonyl stress in tissues.

## Materials and Methods

### Animals

As described in detail previously [13,20], transgenic SHR (hereafter referred to as SHR-CRP) were derived by microinjections of SHR ova with a construct containing cDNA for human CRP under control of the apoE promoter, with the objective of driving expression of the CRP transgene in the liver where CRP is normally produced. To investigate the effects of metformin on inflammation caused by human CRP, we randomized 8-month-old transgenic SHR-CRP into groups with or without metformin treatment. We treated 8-month-old male transgenic SHR-CRP with metformin mixed as part of a standard diet for 4 weeks. The concentration of metformin in the diet was adjusted to deliver a daily metformin dose of approximately 300 mg/kg/day. A corresponding untreated control group of male transgenic SHR-CRP was fed a standard diet without metformin. In a similar fashion, we studied a group of nontransgenic SHR treated with metformin and an untreated group of nontransgenic SHR controls. In each group, we studied 6 animals. The rats were housed in an air-conditioned animal facility and allowed free access to their chow and water. At the end of experiments, animals were sacrificed by cervical dislocation in a postprandial state and tissues were collected for analyses. All experiments were performed using collected tissues only, not live animals. These experiments were performed in agreement with the Animal Protection Law of the Czech Republic and were approved by the Ethics Committee of the Institute of Physiology, Czech Academy of Sciences, Prague (Permit Number: 66/2014**)**.

### Basal- and insulin-stimulated glycogen synthesis in skeletal muscle

In order to measure insulin-stimulated incorporation of glucose into glycogen, diaphragmatic muscles were incubated for 2 hours in 95% O_2_ + 5% CO_2_ in Krebs-Ringer bicarbonate buffer, pH 7.4, containing 0.1 μCi/ml of ^14^C-U glucose, 5 mmol/L of unlabeled glucose and 2.5 mg/ml of bovine serum albumin (Sigma, Fraction V, Czech Republic) with or without 250 μU/ml of insulin. Glycogen was extracted, and insulin-stimulated incorporation of glucose into glycogen was determined.

### Glucose oxidation in muscle

Glucose oxidation was determined *ex vivo* in diaphragmatic muscle by measuring the incorporation of ^14^C-U glucose into CO_2_. Skeletal muscle was immediately incubated for 2 h in Krebs-Ringer bicarbonate buffer, pH 7.4, which contained 5.5 mM of unlabeled glucose, 0.5 μCi/ml of ^14^C-U glucose (UVVR, Prague, Czech Republic) and 3 mg/ml of bovine serum albumin. After 2 h incubation, 0.3 ml of 1M hyamine hydroxide was injected into the central compartment of the incubation vial and 0.5 ml of 1M H_2_SO_4_ was added to the main compartment to liberate CO_2_. The vials were incubated for another 30 min., after which the hyamine hydroxide was quantitatively transferred to the scintillation vial containing 10 ml of toluene-based scintillation fluid for radioactivity counting.

### Glucose utilization in isolated epididymal adipose tissue

Glucose utilization in adipose tissue was determined *ex vivo* by measuring the incorporation of radioactive glucose into adipose tissue lipids. Distal parts of epididymal adipose tissue were rapidly dissected and incubated for 2 hours in Krebs-Ringer bicarbonate buffer with 5 mmol/L glucose, 0.1 μCi ^14^C-U glucose/mL (UVVR, Prague, Czech Republic) and 2% bovine serum albumin, with a gaseous phase of 95% O_2_ and 5% CO_2_ in the presence (250 μU/mL) or absence of insulin in incubation media. All incubations were performed at 37°C in sealed vials in a shaking water bath. Estimation of ^14^C-glucose incorporation into neutral lipids was performed. Briefly, adipose tissue was removed from the incubation medium, rinsed in saline and immediately put into chloroform. The pieces of tissue were dissolved using a Teflon pestle homogenizer, methanol was added (chloroform:methanol 2:1) and lipids were extracted at 4°C overnight. The remaining tissue was removed, KH_2_PO_4_ was added and a clear extract was taken for further analysis. An aliquot was evaporated and reconstituted in scintillation liquid and radioactivity was measured by scintillation counting.

### Lipolysis in isolated epididymal adipose tissue

For measurement of basal and adrenaline stimulated lipolysis, the distal parts of epididymal adipose tissue were incubated in Krebs-Ringer phosphate buffer containing 3% bovine serum albumin (Sigma, Fraction V, Czech Republic) at 37°C, pH 7.4 with or without adrenaline (0.25 μg/ml). The tissue was incubated for 2 hours and the concentrations of NEFA in the medium were determined. Basal lipolysis was measured as NEFA levels after a 2-hour incubation without adrenaline. Stimulated lipolysis was measured as NEFA levels in media after a 2-hour incubation with adrenaline.

### Tissue triglyceride measurements

For determination of triglycerides in liver and soleus muscle, tissues were powdered under liquid N_2_ and extracted for 16 hours in chloroform:methanol, after which 2% KH_2_PO_4_ was added and the solution centrifuged. The organic phase was removed and evaporated under N_2_. The resulting pellet was dissolved in isopropyl alcohol, and triglyceride content was determined by enzymatic assay (Erba-Lachema, Brno, Czech Republic).

### Biochemical analyses

Rat serum CRP and human serum CRP were measured using ELISA kits (Alpha Diagnostics International, San Antonio, U.S.A.). Blood glucose levels were measured by the glucose oxidase assay (Erba-Lachema, Brno, Czech Republic) using tail vein blood drawn into 5% trichloroacetic acid and promptly centrifuged. NEFA levels were determined using an acyl-CoA oxidase-based colorimetric kit (Roche Diagnostics GmbH, Mannheim, Germany). Serum triglyceride concentrations were measured by standard enzymatic methods (Erba-Lachema, Brno, Czech Republic). Serum insulin concentrations were determined using a rat insulin ELISA kit (Mercodia, Uppsala, Sweden). Serum IL-6 and TNFα were measured using rat ELISA kits (BioSource International, Inc., Camarillo, U.S.A.). Serum MCP-1 was determined using kit from eBioscience, Bender MedSystems Biocenter, Vienna, Austria.

### Parameters of oxidative stress

The activity of superoxide dismutase (SOD) was analyzed using the reaction of blocking nitrotetrazolium blue reduction and nitroformazan formation. Catalase (CAT) activity measurement was based on the ability of H_2_O_2_ to produce with ammonium molybdate a color complex detected spectrophotometrically. The activity of seleno-dependent glutathione peroxidase (GSH-Px) was monitored by oxidation of gluthathione using Ellman reagent (0.01М solution of 5,5'-dythiobis-2 nitrobenzoic acid). The level of reduced glutathione (GSH) was determined in the reaction of SH-groups using Ellman reagent. Glutathione reductase (GR) activity was measured by the decrease of absorbance at 340 nm using a millimolar extinction coefficient of 6220 M^-1^cm^-1^ for NADPH (using the Sigma assay kit). Lipoperoxidation products were assessed according to levels of thiobarbituric acid reactive substances (TBARS) determined by assaying the reaction with thiobarbituric acid.

### Parameters of dicarbonyl stress

Levels of reactive dicarbonyl methylglyoxal (MG), glyoxal (GL) and 3-deoxyglucosone (3-DG) in serum and tissues were determined by high performance liquid chromatography. After derivatization with diamino-dimethoxybenzene, samples were injected in the C18 column (Waters Corporation) over mobile phase and detected by fluorescence [21].

### Histology

The heart was fixed in 10% buffered formalin. The heart was transverally cut up at one third of lenght from its apex. Samples were processed by the common paraffin technique and histological slices 5 μm thick were stained with hematoxylin and eosine.

### Gene expression profiles

Total RNA was extracted from the livers of SHR-CRP transgenic and SHR control rats (N = 4 per group) treated with metformin or with placebo. The quality and concentration of RNA were determined using a NanoDrop 2000 spectrophometer (Thermo Scientific). RNA integrity was analyzed in an Agilent Bioanalyzer 2100. We only included samples judged to have an intact RNA profile. The Affymetrix GeneChip^®^ Rat Gene 1.0 ST Array System was used for the microarray analysis following the standard protocol: 100 ng RNA was amplified with an Ambion WT Expression Kit (Applied Biosystems), 5.5 μg single-stranded cDNA was labeled and fragmented with GeneChip WT Terminal Labeling and Hybridization (Affymetrix) and hybridized on the chip according to the manufacturer’s procedure. The analysis was performed in three replicates. The transcription data were MIAME-compliant and deposited in the ArrayExpress database (ID # E-MTAB-3791).

### Gene expression determined by real time PCR

Total RNA was extracted from the liver using Trizol reagent (Invitrogen), and cDNA was prepared and analyzed by real-time PCR testing using QuantiTect SYBR Green reagents (Qiagen, Inc.) on an Opticon continuous fluorescence detector (MJ Research). Gene expression levels were normalized relative to the expression of the peptidylprolyl isomerase A (*Ppia*) (cyclophilin) gene, which served as the internal control, with results being determined in triplicate. Primers used for the validation of differentially expressed genes selected from significant pathways are given in S1 Table.

### Statistical analysis

Our goal was to determine whether the anti-inflammatory effects of metformin treatment would be greater in the transgenic strain (expressing human CRP) than in the nontransgenic strain. To this end, we used 2 way ANOVA to test for treatment x strain interactions and, if present, we determined whether metformin inhibited inflammation to a greater extent in the SHR-CRP transgenic strain than in the nontransgenic strain. For variables showing evidence of treatment x strain interaction we used the Holm-Sidak test, which adjusts for multiple comparisons to determine whether the anti-inflammatory effects of metformin were significant in the nontransgenic SHR strain and in the transgenic SHR-CRP strain expressing human CRP. Results are expressed as means ± S.E.M.

Gene expression data were preprocessed using Partek Genomic Suite (Partek Incorporated) software. Analyses were performed using methods similar to those previously described [22]. Briefly, the transcription profiles were background-corrected using the RMA method, probe sets summarized using median polish, quantile normalized and variance-stabilized using base-2 logarithmic transformation. Analysis of variance yielded transcripts differentially expressed between analyzed samples (within LIMMA) [23]. Storey’s q values [24] were used to select significant and differentially expressed genes (q<0.05). All statistical analyses were performed in R and under Bioconductor [25]. To identify significantly perturbed pathways, we performed SPIA (Signaling Pathway Impact Analysis) [26] on KEGG pathways: genes with P<0.05 were considered differentially transcribed. Where appropriate, the resulting statistical p-values were corrected for multiple testing using the FDR method [27].

## Supporting Information

S1 Fig **Validation of gene expression profiles obtained by Affymetrix transcriptional profiling using quantitative real time PCR for four transcripts in livers isolated from (A) SHR-CRP untreated rats (open bars) versus SHR-CRP treated with metformin (solid bars), or from (B) SHR untreated rats (open bars) versus SHR treated with metformin (solid bars).** Expression of selected genes was normalized relative to the expression of the peptidylprolyl isomerase A (*Ppia*) gene, which served as an internal control. * and ** denote p<0.05 and p<0.005, respectively.
(PPT)

S1 Table Primers for validation of directional expression of genes identified by gene expression profiling. (DOC)

S2 Table LIMMA analysis (contrast metformin versus placebo of 51 genes with q-value < 0.05). Rows in light red indicate upregulation, FDR is not significant; rows in dark red indicate upregulation, FDR is significant (q<0.05); rows in light blue indicate downregulation, FDR is not significant; rows in dark blue indicate downregulation, FDR is significant (q<0.05).
(XLS)
