# Supplementary figures and images for: Effects of Metformin on Tissue Oxidative and Dicarbonyl Stress in Transgenic Spontaneously Hypertensive Rats Expressing Human C-Reactive Protein
Source: PLoS One. 2016 Mar 10;11(3):e0150924. doi: 10.1371/journal.pone.0150924 (PMC4786274; doi:10.1371/journal.pone.0150924)

## Slide 1
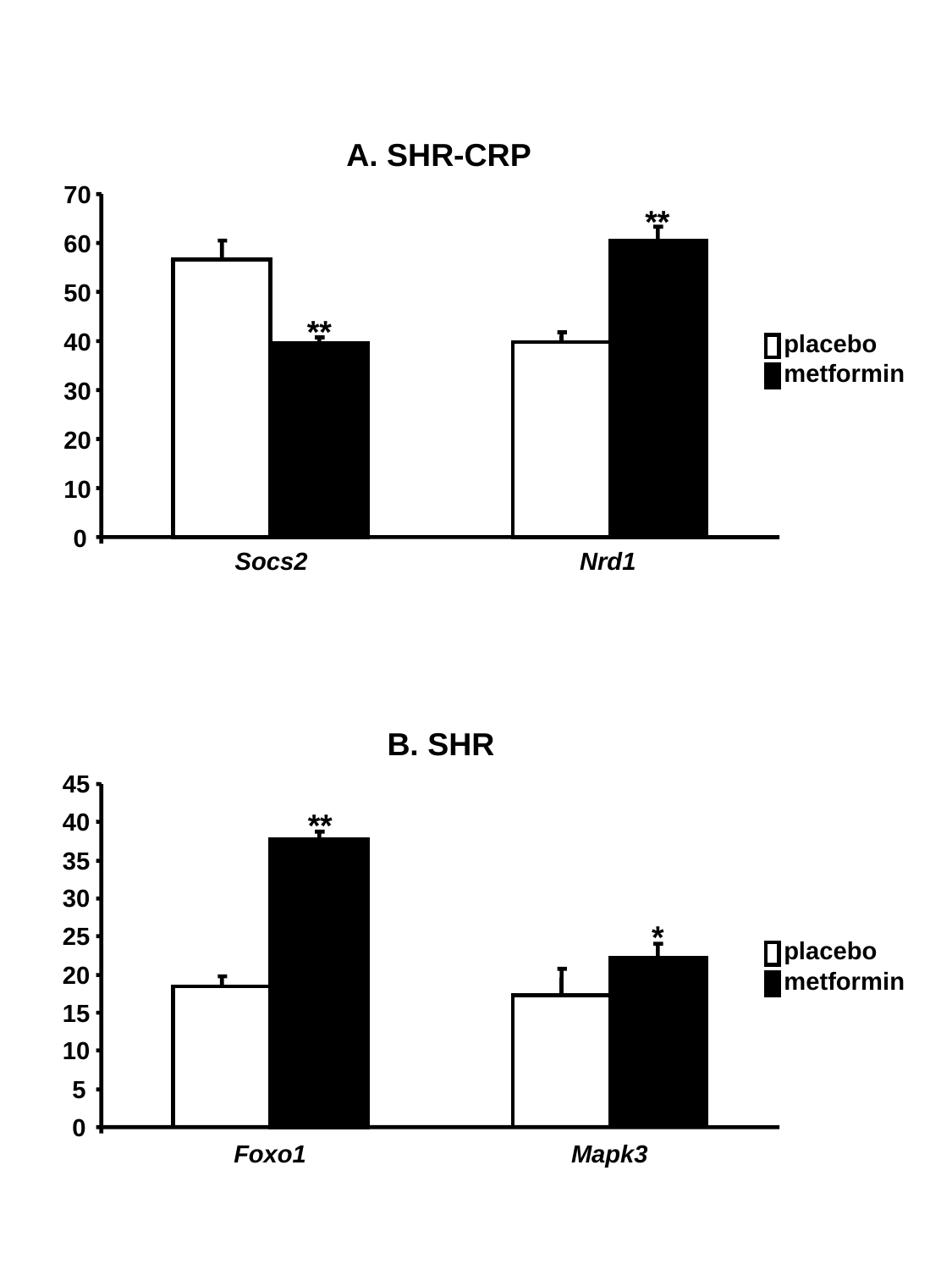

A. SHR-CRP
70
**
60
50
**
40
placebo
metformin
30
20
10
0
Socs2
Nrd1
B. SHR
45
**
40
35
30
*
25
placebo
20
metformin
15
10
5
0
Foxo1
Mapk3

Supplement: S1 Fig — Validation of gene expression profiles obtained by Affymetrix transcriptional profiling using quantitative real time PCR for four transcripts in livers isolated from (A) SHR-CRP untreated rats (open bars) versus SHR-CRP treated with metformin (solid bars), or from (B) SHR untreated rats (open bars) versus SHR treated with metformin (solid bars). Expression of selected genes was normalized relative to the expression of the peptidylprolyl isomerase A (Ppia) gene, which served as an internal control. * and ** denote p<0.05 and p<0.005, respectively. (PPT) [file pone.0150924.s001.ppt]
