# Supplementary material for: Effects of Metformin on Tissue Oxidative and Dicarbonyl Stress in Transgenic Spontaneously Hypertensive Rats Expressing Human C-Reactive Protein
Source: PLoS One. 2016 Mar 10;11(3):e0150924. doi: 10.1371/journal.pone.0150924 (PMC4786274; doi:10.1371/journal.pone.0150924)
Supplement: S1 Table — (DOC) [file pone.0150924.s002.doc]

**Supplementary Table 1** Primers for validation of directional expression of genes identified by gene expression profiling.

| **Genes** | **Forward primers** | **Reverse primers** |
| --- | --- | --- |
| *Foxo1* | AGGAGTTAGTGAGCAGGCAAC | GGGTGAAGGGCATCTTTGGA |
| *Mapk3* | CACACTGGCTTTCTGACCGA | TGGATTTGGTGTAGCCCTTGG |
| *Nrd1* | ACGGGGCGAGAGAGGG | ACACCACCTGTGTTGTTGTTG |
| *Ppia* | agcatacaggtcctggcat | tcaccttcccaaagaccac |
| *Socs2* | GTGAGCTCAGTCAAACAGGATG | TTCCTTCTGGCGCCTCTTTTA |
